# CIFAR10-DVS: An Event-Stream Dataset for Object Classification

**DOI:** 10.3389/fnins.2017.00309

**Published:** 2017-05-30

**Authors:** Hongmin Li, Hanchao Liu, Xiangyang Ji, Guoqi Li, Luping Shi

**Affiliations:** ^1^Department of Precision Instrument, Center for Brain-Inspired Computing Research, Tsinghua UniversityBeijing, China; ^2^Department of Automation, Tsinghua UniversityBeijing, China

**Keywords:** neuromorphic vision, event-based vision, dynamic visions sensor (DVS), address event representation, frame-free vision

## Abstract

Neuromorphic vision research requires high-quality and appropriately challenging event-stream datasets to support continuous improvement of algorithms and methods. However, creating event-stream datasets is a time-consuming task, which needs to be recorded using the neuromorphic cameras. Currently, there are limited event-stream datasets available. In this work, by utilizing the popular computer vision dataset CIFAR-10, we converted 10,000 frame-based images into 10,000 event streams using a dynamic vision sensor (DVS), providing an event-stream dataset of intermediate difficulty in 10 different classes, named as “CIFAR10-DVS.” The conversion of event-stream dataset was implemented by a repeated closed-loop smooth (RCLS) movement of frame-based images. Unlike the conversion of frame-based images by moving the camera, the image movement is more realistic in respect of its practical applications. The repeated closed-loop image movement generates rich local intensity changes in continuous time which are quantized by each pixel of the DVS camera to generate events. Furthermore, a performance benchmark in event-driven object classification is provided based on state-of-the-art classification algorithms. This work provides a large event-stream dataset and an initial benchmark for comparison, which may boost algorithm developments in even-driven pattern recognition and object classification.

## Introduction

Neuromorphic vision based on neuromorphic cameras (Delbruck, 2008; Lichtsteiner et al., 2008; Serrano-Gotarredona and Linares-Barranco, 2013; Yang et al., 2015) which converts visual information to address-event-representation (AER; Boahen, 2000) event streams has garnered increasing attention. For the pattern recognition and object classification, high-quality datasets provide challenging benchmarks to support the continuous development and comparison of sophisticated and robust algorithms and methods (Sotiris et al., 2006). In computer vision, a number of well-established datasets i.e., CIFAR-10 (Krizhevsky, 2009), MNIST (Lecun et al., 1998), and Caltech101 (Fei-Fei et al., 2007) are available for researchers. In addition, they can obtain the frame-based images or create frame-based datasets on the internet or by using digital cameras. Although many high-quality datasets have promoted the development of the computer vision field (Tan et al., 2015), a very limited number of datasets are available for the researchers in neuromorphic vision. Contrary to generating conventional computer vision datasets, it is very difficult to develop a neuromorphic vision dataset by collecting data online. Thus, creating datasets for neuromorphic vision researchers is an important yet challenging task. Generally, a high-quality event-stream classification dataset should provide a challenge for the state-of-the-art event-based algorithms. It needs to be difficult enough to prevent the current algorithms from achieving near perfect accuracy even in the face of significant algorithmic optimization. But, the challenge should not be too difficult to allow any significant improvements to achieve a relatively good accuracy.

Converting an existing frame-based dataset into an event-stream version saves time without trying to find a number of objects of different classes to record and label. Using popular image datasets enables an easier comparison between the two communities. Recently, two popular image datasets have been converted into event-stream datasets. One is the MNIST dataset which was converted into two neuromorphic vision datasets, i.e., MNIST-DVS and N-MNIST (Orchard et al., 2015; Serrano-Gotarredona and Linares-Barranco, 2015). In the MNIST-DVS dataset, 10,000 digits were chosen from the standard 70,000-picture dataset and displayed on an LCD monitor at three different scales for about 2–3 s. In conversion of the N-MNIST dataset, all the digits of MNIST were converted into the event streams with an asynchronous time-based image sensor (ATIS). There are few background noise events because all the digits of MNIST are placed on a uniform background. As is known, high accuracy rate has been achieved on the two neuromorphic versions of MNIST dataset (Orchard et al., 2015; Zhao et al., 2015). The other is the Caltech101 dataset which contains 100 image classes with all converted (Orchard et al., 2015). The natural images of Caltech101 are more complicated than the digits of MNIST. Being more challenging, 100 different categories of the dataset make it more difficult for the improvement and optimization of current event-driven algorithms. However, although the event-stream versions of the two image datasets have provided good benchmarks in neuromorphic vision, a neuromorphic dataset of moderate difficulty with less object classes than N-Caltech101 and more complicated objects than digits is still lacking. CIFAR-10 is a more complicated frame-based image dataset than MNIST while it has fewer categories than the Caltech101. Current state-of-the-art classification accuracy for frame-based algorithms on CIFAR-10 is 96.53% (Springenberg et al., 2015). In this paper, the CIFAR-10 is converted into a moderate-level neuromorphic vision dataset in 10 classes.

There are two ways that are usually used in the conversion of neuromorphic data. One way is to convert the frame-based images into event streams by simulation (Masquelier and Thorpe, 2007; O'Connor et al., 2013). Simulation is not completely equal to the realistic recording with an event-based camera. If an image is captured with a frame-based camera, then the temporal information is inherently lost. Besides, noise in the real world is not presented in simulation. The second way is to generate the event streams with a neuromorphic camera (Orchard et al., 2015; Serrano-Gotarredona and Linares-Barranco, 2015). Garrick Orchard (Orchard et al., 2015) employed the camera movement of saccade by developing an actuated pan-tilt platform to move an ATIS camera in front of a LCD monitor. Compared with the camera movement, image movement (Serrano-Gotarredona and Linares-Barranco, 2015) is easier to control. And in addition, the image movement is closer to practical applications than the pan-tilt method. For example, the neuromorphic cameras are suitable for monitoring pedestrians or other moving objects.

In this paper, we created an event-steam dataset named “CIFAR10-DVS” by converting an popular frame-based image dataset “CIFAR-10” using a DVS camera (Lichtsteiner et al., 2008). The camera has the spatial resolution of 128 × 128. In the dataset name “CIFAR10-DVS,” “DVS” represents the DVS camera as the same as MNIST-DVS. The CIFAR-10 dataset consists of 60,000 32 × 32 color images in 10 classes, with 6,000 images per class. In the conversion, 1,000 images per class were selected which is the same to MNIST-DVS. The converted event streams of the CIFAR-10 images have more complicated spatio-temporal structures than MNIST-DVS. The fewer categories make the created CIFAR10-DVS simpler than N-Caltech101. Therefore, the CIFAR10-DVS is a moderate-level event-stream dataset providing space for continuous improvements of event-driven algorithms. Image movement is employed to produce the intensity changes within the visual field of the event-based camera. When the change of local log intensity exceeds a pre-defined threshold, an event is generated. Unlike the camera movement method, the image movement is more easily implemented by programing on computers and closer to practical applications. In this work, a repeated closed-loop smooth (RCLS) movement of image was used to produce the rich oriented gradients of intensity captured by the neuromorphic camera. This work provides a large event-stream dataset and an initial benchmark for comparison, which may boost the event-driven algorithm developments in object recognition of neuromorphic vision.

The remainder of this paper is divided into four parts. Section Materials and Methods presents the recording system consisting of hardware system and software system. Section Data Properties presents the statistical properties of the CIFAR10-DVS dataset. In Section Performance Metric, the recognition accuracies of the existing algorithms are presented before wrapping up with conclusion in Section Conclusion.

## Materials and methods

In this section, the principle and methodology of converting the event-stream dataset is introduced. No complex hardware modules are required in our recording method. In Section Repeated Closed-Loop Smooth (RCLS) Movement, the RCLS movement is presented which is the core of our approach. Section Recording System introduces the hardware and software modules of the recording system. The proposed dataset can be accessed online^1^.

### Repeated closed-loop smooth (RCLS) movement

**Figure 1:**
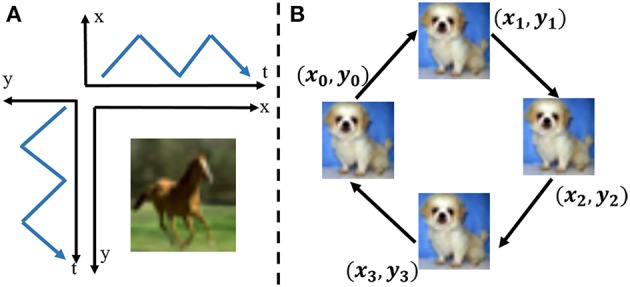
**(A)** Image movement along x axis and y axis simultaneously over time in the 2D intensity field. The moving image generates the local intensity changes in both x direction and y direction at the same time. **(B)** The RCLS movement of an image. Four paths make up the closed-loop movement. Each path is at the angle of 45°.

In each DVS recording, the event cluster forms the shape of the object. Generally, for a specified image, more events except the surrounding noise events make the spatio-temporal shape of the object clearer. To generate more events, more local intensity changes should be captured by the event camera. In this work, the RCLS image movement is used to convert the oriented gradients of the image into the local relative intensity changes in the view field of the DVS camera. Because of the response time of the neuromorphic device, a smooth movement at an appropriate speed is beneficial to generate more events. In fact, the smooth movement is also associated with human vision because we cannot sense the leaping objects very well due to the limited speed of visual processing (Thorpe et al., 1996). The DVS camera is specifically designed such that the pixel responses to the temporal contrast of intensity and generates an event whenever the contrast exceeds a threshold contrast. The repeated closed-loop image movement in continuous time generates rich local intensity changes and each pixel of the DVS camera continuously quantizes the local relative intensity changes to generate events. The event rate or event number during a time interval is proportional to the temporal contrast which is defined as

(1) $$TCON=\frac{d(ln(I(x,y)))}{dt}=\frac{1}{I(x,y)}\frac{dI(x,y)}{dt}$$

where *I(x, y)* demotes brightness at the *(x, y)* position of the view field of the neuromorphic camera. Suppose that the view field of the DVS camera is a 2D intensity field and the frame-based image is located in the intensity field as shown in Figure 1A. Each pixel value denotes the intensity of the corresponding position in the intensity field. The image movement converts the intensity gradients of the image to the intensity changes over time in the intensity field. Each pixel independently and in continuous time quantizes the relative intensity changes of the corresponding point in the intensity field to generate events. The intensity changes produced by the closed-loop movement are shown in Figure 1B. The closed-loop movement consists of four paths which mimic the saccade of the biological vision. Intensity gradients of two directions are captured by the neuromorphic camera synchronously. For each point in the intensity field, the intensity change over time is represented as follows,

(2) $$\frac{dI(x,y)}{dt}=\frac{dI(x,y)}{dx}\cdot \frac{dx}{dt}+\frac{dI(x,y)}{dy}\cdot \frac{dy}{dt}=I_{x}\cdot V_{x}+I_{y}\cdot V_{y}$$

where *I*_*x*_, *I*_*y*_ represent the intensity gradients of the image with respect to *x* and *y* spatial co-ordinates on the 2D intensity field respectively. *V*_*x*_ and *V*_*y*_ are the velocities of the moving image in the *x* and *y* directions. The Equation (2) describes how the image movement results in the intensity change of the intensity field.

Combing Equations (1,2), the relationship between temporal contrast and image movement can be described as

(3) $$TCON=\frac{1}{I\left(x,y\right)}\left(I_{x}\cdot V_{x}+I_{y}\cdot V_{y}\right)=I_{x}^{ln}V_{x}+I_{y}^{ln}V_{y}$$

where $I_{x}^{ln}=\frac{d\left(\text{l}\text{n}\left(I\left(x,y\right)\right)\right)}{dx}$ and $I_{y}^{ln}=\frac{d\left(\text{l}\text{n}\left(I\left(x,y\right)\right)\right)}{dy}$ denote the derivatives of logarithmic intensity field on the pixel with respect to x and y, respectively. Then images with edges of higher contrast tend to result in more events in their event-stream recordings. Because of the response time of the device, the movement should not be too fast.

### Recording system

A recording system which implements the RCLS movement is developed. A LCD monitor is used to display the moving images. The recording system contains both hardware system and software system. The core of the hardware system is the DVS camera which converts the moving objects into event streams. The bias values of the used DVS camera are shown in Table 1. The camera sits on a bracket which makes the middle of the sensor a high of 250 mm to line up with the vertical center of the monitor. A monitor is connected with a host PC. Different from Orchard et al. (2015), our recording system need no motors and control circuit. The recording system is placed in a dark location to suppress any stray light of the environment.

**Table 1:** Bias values of the used DVS camera.

**Name of bias**	**Value**	**Brief description**
Pr	13.5 p	First stage (“Photoreceptor”)
cas	242.5 p	First stage cascode
foll	229.0 p	Source follower separating first and second stages
diff	135.4 n	Second stage (“Differential”)
diffOn	2.2 u	Threshold for On events
diffOff	592.7 p	Threshold for Off events
injGnd	5.0 u	Injected ground
req	714.6 n	Pull down for passive load inverters in digital AER pixel circuitry
Refr	26.9 p	Refractory period
PuX	75.3 u	Pull up on request from X arbiter
PuY	36.6 u	Pull up on request from Y arbiter
reqPd	75.3 u	Pull down on chip request

**Figure 2:**
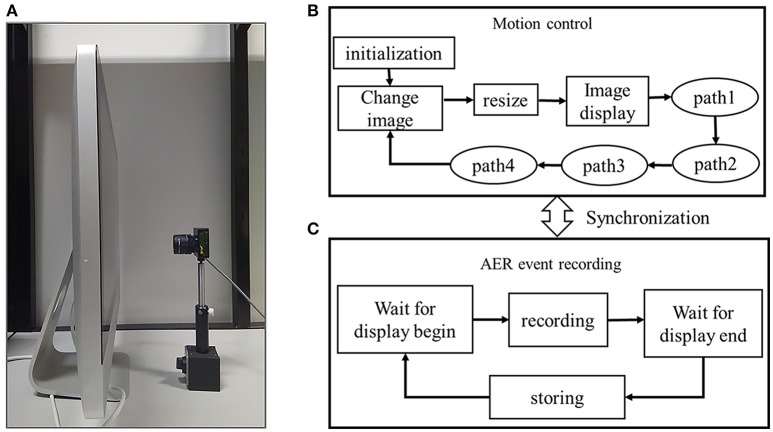
**(A)** Hardware system of the event stream recording system. A DVS camera is placed viewing the LCD monitor. **(B)** Image movement control and display part, **(C)** AER event stream recording part.

The software system running on the host PC contains an image display component and an AER recording component of the DVS output, as shown in Figure 2. The image display component is based on Python programming language. The python version of CIFAR-10 dataset is downloaded and each color image is constructed from the three RGB matrixes. The image display component contains an image display program and an image movement control program. The image display program displays each image in a window. The display window is the so-called intensity field. In the display window, each image moves in order to generate the intensity changes. During the initialization, the selected CIFAR-10 images are loaded and constructed. The movement parameters are set and the display window are created and located on an appropriate position of the monitor so that the DVS camera can cover the maximum possible view field on the display window. During the image change, a new image is constructed from the dataset after 2,000 ms since the image movement is over so that the next recording will not be influenced by the last image movement. Besides, the current recording is stored during this process. During the image resizing process, an image is upsampled from original 32 × 32 to 512 × 512 using bicubic interpolation. The recordings will be very fuzzy when low-resolution and tiny images are displayed on the monitor. Through resizing, the edges and textures of the images are magnified to better fit into the visual field of the DVS camera. Each closed loop contains four moving paths. During the path 1, path 2, path 3, path 4 processes, the upscaled image moves uniformly at a certain speed. During each image movement, the closed loop is repeated several times (i.e., 6 times). After the *path 4* of the final circle, display of the image is over and a message is sent to the AER recording program. The direction of each path is not strictly horizontal or vertical, but at an angle of 45°, which ensures that intensity gradients of two directions are captured synchronously.

The AER recording component is based on jAER (Delbrück, 2006), an open source software interface between DVS camera and host PC provided by Tobi Delbrück's group. In our recording program, an inter-process communication with the image movement control program is implemented to synchronize the displaying of images and recording of the event streams. When a new image is display on the LCD monitor, the recording program starts to capture the AER events. When the closed-loop movement of an image is over, the process will store the event stream automatically.

Table 2 shows the dynamics of the image movement. We discretized each path in five segments using four internal points in order to realize the smooth movement. At each point, there will be an image shown for about 10 ms which is fast than the screen refresh of 60 Hz. Then on each path, the moving image will be captured at one of the five points after screen refresh, which adds some randomness to the recordings as well. Each recording takes about 3,200 ms to be captured (1,200 ms of the closed-loop movement plus 2000 ms for transition between images).

**Table 2:** Parameters of image movement control component during the closed-loop movement.

**State**	**Start time**	**Start (pixel)**		**End (pixel)**		**Speed (pixel/s)**	
	**(ms)**	**X**	**Y**	**X**	**Y**	**X**	**Y**
Change and resize image	2,000	0	0	0	0	0	0
Path 1	50	0	0	10	−10	200	−200
Path 2	100	10	−10	20	0	200	200
Path 3	150	20	0	10	10	−200	200
Path 4	200	10	10	0	0	−200	−200

The original CIFAR-10 consists of 60,000 color images in 10 classes, with 6,000 images per class. In our conversion, 10,000 images are randomly selected from the CIFAR-10 with 1,000 images per class which is the same with the MNIST-DVS. The second reason of recording one-sixth of the original frame-based image dataset is the huge size of the recordings in storage.

## Results

### Data properties

Hidden statistical properties have an important influence on the performance of event-driven classification algorithms. All the recordings of the CIFAR10-DVS were analyzed to explore some of the basic statistical properties (i.e., the mean and standard deviation of the ON and OFF events, the mean and deviation of the x- and y- addresses, the mean and deviation of the x and y range) as shown in Table 3. Other neuromorphic vision datasets, i.e., MNIST-DVS^2^, N-MNIST & N-Caltech101^3^, were also analyzed to check the same statistical properties for comparison. Unlike other event-stream datasets, in CIFAR10-DVS dataset, there are relatively more OFF events than ON events. This may result from the fact that the CIFAR-10 images have complex objects and backgrounds which more easily boost OFF events. Besides, event-based sensors are almost impossible to produce the same number of ON and OFF events. The properties of the used DVS camera may be more sensitive to the negative threshold events. Both the mean x-addresses and y-addresses depend on the image size and the image content, which is a good feature for classification. The ratio of number of ON events to OFF events depends on the different objects, which can subsequently be used as a feature for classification.

**Table 3:** Statistical properties of four neuromorphic vision datasets.

**Dataset**	**CIFAR10-DVS**		**MNIST-DVS**		**N-MNIST**		**N-Caltech101**	
**Statistic**	**Mean**	**σ**	**Mean**	**σ**	**Mean**	**σ**	**Mean**	**σ**
ON events	86,551	29,182	36,522	934.04	2,084	574	56,936	28,039
OFF events	11,852	23,815	37,219	964.22	2,088	623	58,180	30,021
X mean	67.24	11.46	62.11	2.43	17.66	5.05	100.72	57.78
Y mean	63.84	6.08	64.28	2.78	18.10	6.38	81.23	46.15
X range	126.99	0.02	126.99	0.12	34.00	0.00	198.53	43.08
Y range	127	0	125.99	0.28	34.00	0.00	155.88	26.76

**Figure 3:**
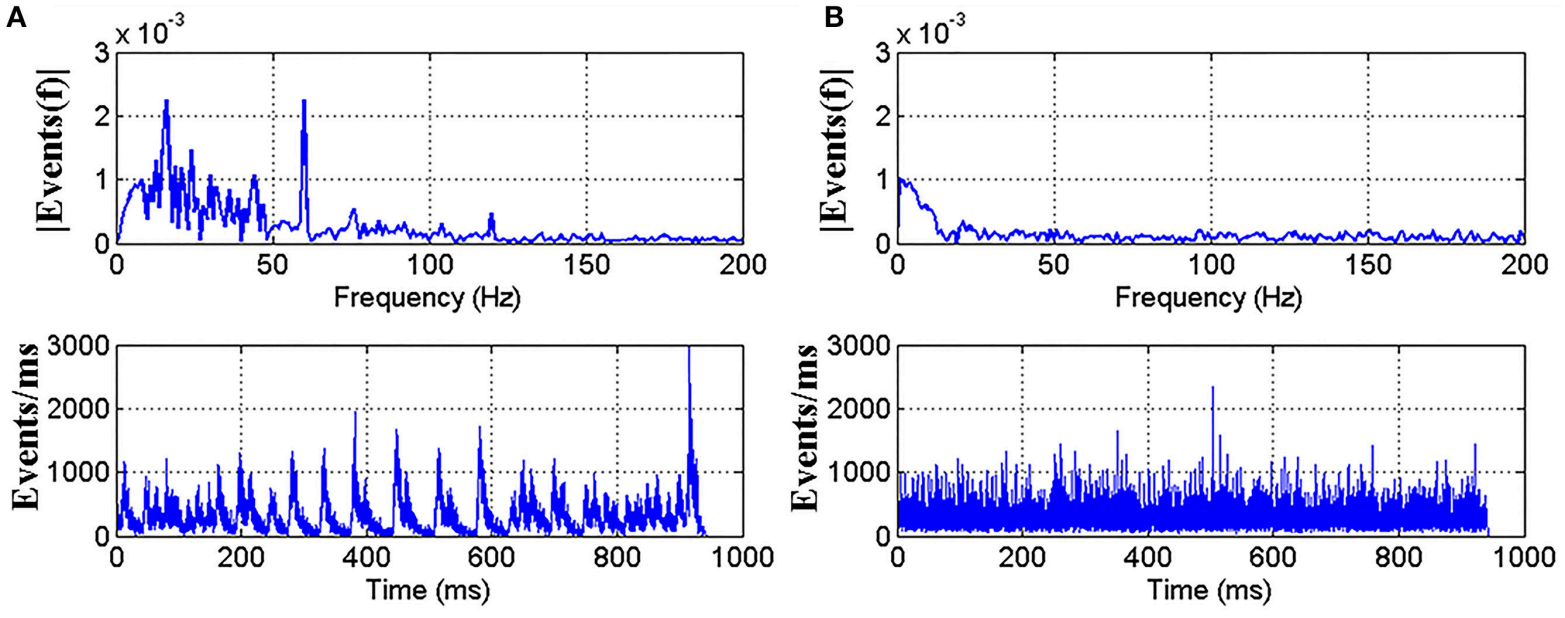
A Fourier analysis showing the impact of LCD screen refresh rate. **(A)** Top shows the timestamp sequence spectrum of the recorded AER events with a clear peak at 60 Hz. The mean inter-spike difference is 3.20 us and standard deviation is 5.24 us. Bottom shows the firing rate curve of the event stream. **(B)** Top shows the spectrum of the same recording after readjusting the timestamps to remove the 60 Hz peak. Timestamps are generated randomly with the same mean inter-spike time difference and standard deviation as in **(A)**.

A spectral analysis on the timestamp sequence of a recording is performed as shown in Figure 3A. A clear peak at 60 Hz introduced by the 60 Hz LCD screen refresh rate comes out of the noise floor. In Serrano-Gotarredona and Linares-Barranco (2015), a adjusting method is proposed to remove the refresh noise. In this work, the adjusting method is used to remove the refresh noise of our datasets. Figure 3B shows the spectrum of the resulting re-timed sequence, where the 60 Hz peak has been removed. In addition, multiple low-frequent peaks also disappeared, which is a part of the adjustment process.

**Figure 4:**
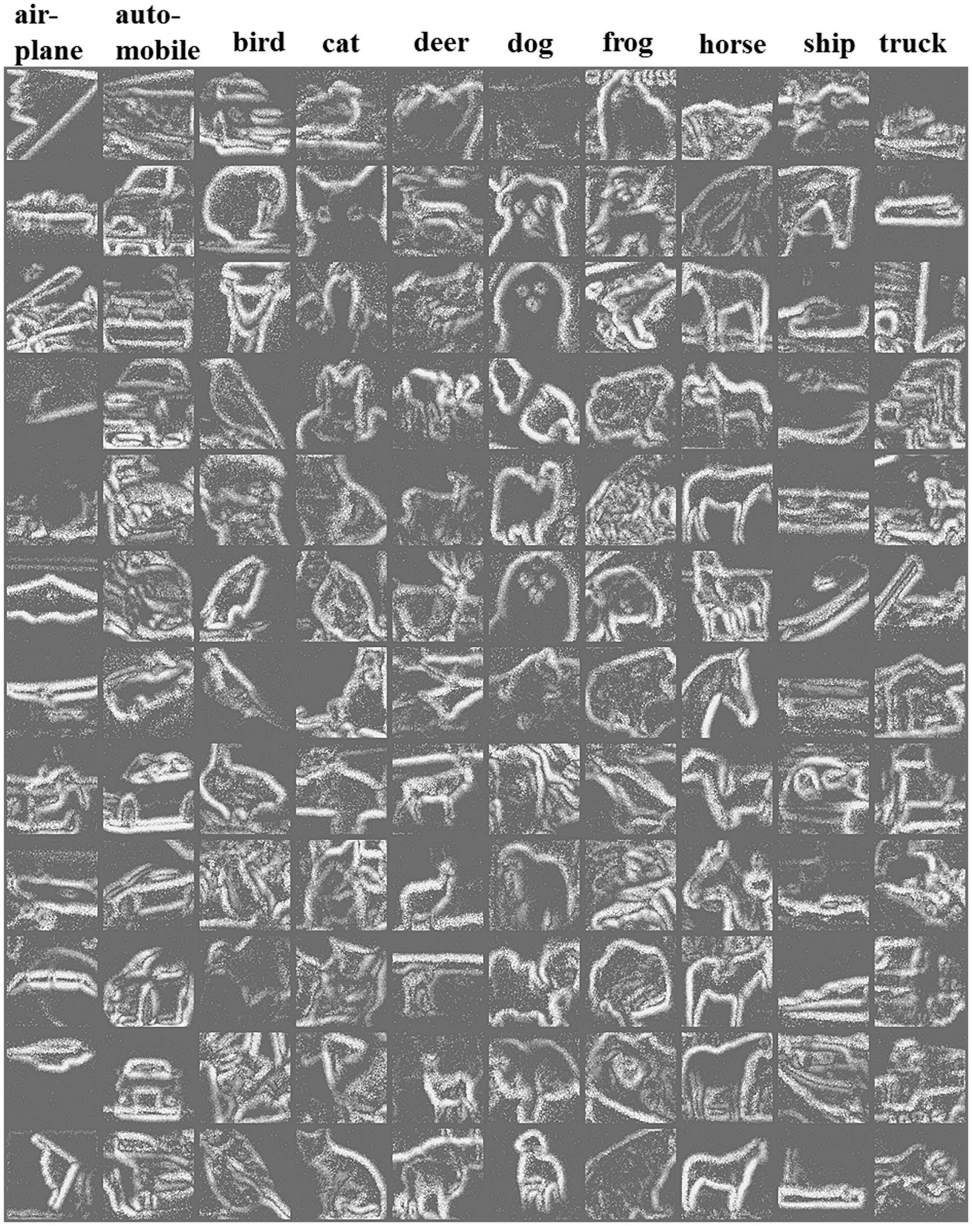
A 12 × 10 matrix of reconstructed frames of randomly selected recordings in CIFAR10-DVS. From left to right columns are AER recordings of 10 categories of airplane, automobile, bird, cat, deer, dog, frog, horse, ship, truck, respectively. In the reconstruction, events over a certain time range are integrated into the corresponding pixel values.

**Figure 5:**
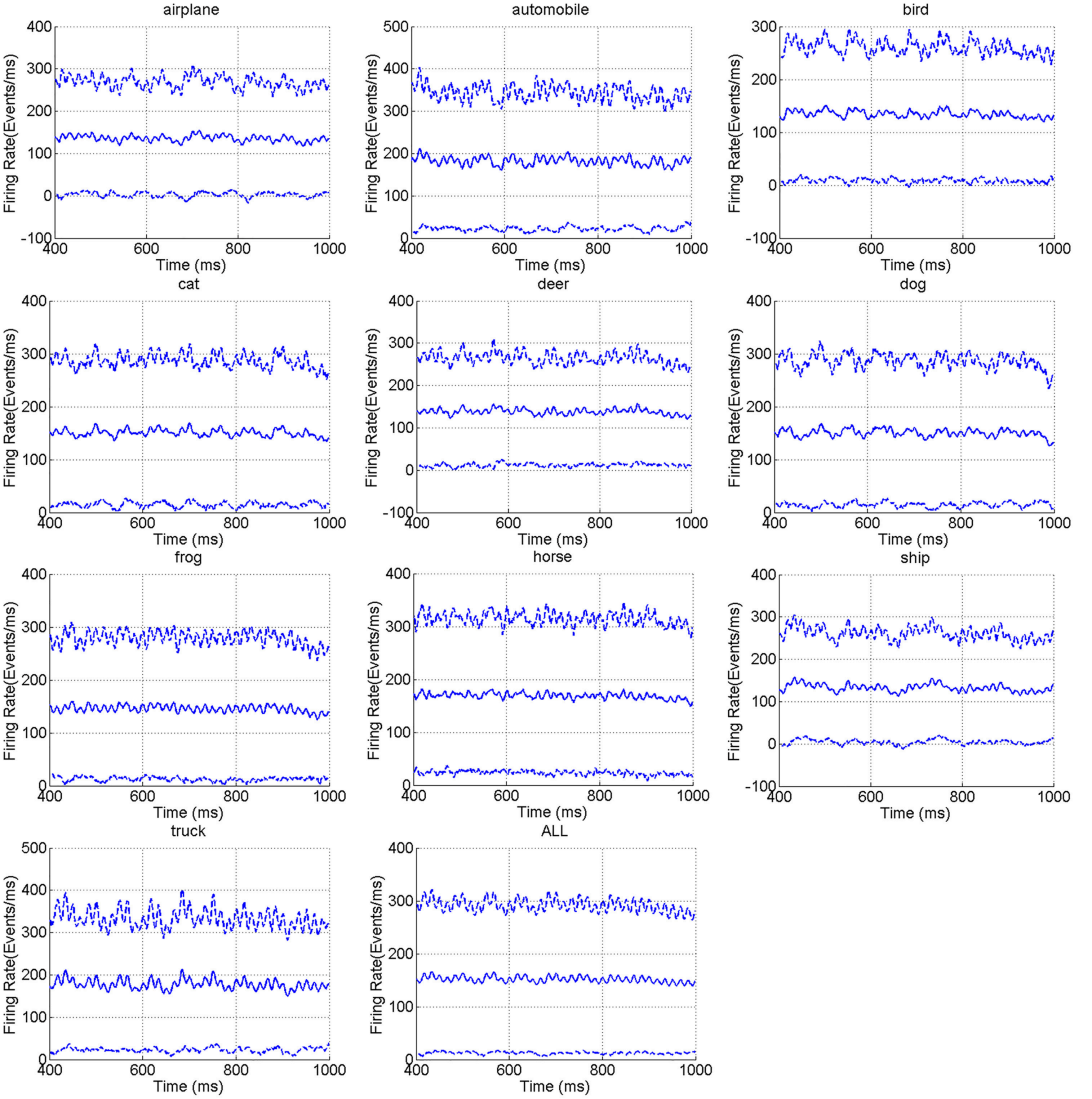
The mean firing rate (solid line) and mean ± standard deviation firing rate (dotted line) for the CIFAR10-DVS categories.

Figure 4 shows the reconstructed frames of 12 randomly selected example recordings per category. The reconstruction is done in the time range of 100 ms. Due to the different properties of edges and contrast of different images of CIFAR-10, some recordings are clear in the reconstructed frames, while some are a little blurry. Figure 5 shows the mean firing rate across time for overall categories in CIFAR-10. It is observed that the mean firing rate of each category is around a particular constant with slight periodic perturbation. The standard deviation of the firing rate is a large value. This phenomenon may result from the diversity of the intensity gradients of the images of the original CIFAR-10 dataset. In the conversion process, each image generates a different number of events, which results in diversity of the firing rates.

### Performance metric

Many event-driven classification algorithms have been proposed to solve the classification task of the DVS recordings (Chen et al., 2012; Zhao et al., 2015; Peng et al., 2016). Here the classification results using two state-of-the-art algorithms are briefly presented to provide an initial classification benchmark. Similar to Orchard et al. (2015), these algorithms were used without any modification as not to put the focus on the tuning and optimization of the algorithms in this work. The algorithms include a spike-based forward network (Zhao et al., 2015), and support vector machine (SVM) classifier using bag of events (BOE) feature (Peng et al., 2016). In addition, K-Nearest Neighbor (KNN) classifier with eight statistical properties was used to provide the initial results for comparison. The detailed description of each algorithm can be found in the following algorithm descriptions.

#### Classification by statistics

The statistical properties of the event streams can be used as features for classification. A KNN classifier with *k* = 10 was used to predict the class of each recording. Ninety percent of the recordings were randomly selected as the training set, and the rest were used as the testing set. Eight statistical properties, i.e., Number of events (NoE), Number of ON events (NoONE), Number of OFF events (NoOFFE), Ratio of positive events to negative events (RoPN), Mean of X address (MNoXA), Mean of Y address (MNoYA), Standard deviation of X address (SDoXA), Standard deviation of Y address (SDoYA), were used as the feature for classification. In addition, all the statistics were combined into a feature vector and used for classification. Ten-fold cross validations were carried out. The mean and standard deviation of the 10 classification results for each statistical feature were calculated for comparison.

#### Spiking forward neural network (SFNN)

The event-driven neural network (Zhao et al., 2015) is a two-step (feature extraction and classifier design) classification method. Bio-inspired cortex-like features are extracted using a convolution-based network of a hierarchy of two layers (S1 and C1). In S1 layer, the event-driven convolution with a forgetting mechanism is introduced for continuously event-driven processing. Neurons of S1 layer compete with local neighbors through the MAX operation to strive for survival in C1 layer. In C1 layer, the survived neurons represent some salient bar features. Furthermore, a motion symbol detector module consisting of a leaky integration neuron and a peak detection unit is introduced to accumulate the events into a pattern. The data flow of the feature extraction stage in the SFNN system can be summarized as *motion events*→*S1 maps*→*C1 maps*. S1 and C1 maps are updated for each incoming spike. A network of Gabor filters with each filter modeling a certain size of receptive field is used to convolve the input events. In our paper, the filter network has four scales (ranging from three to nine with a step of two) and four orientations (0°, 45°, 90°, 135°). For the classification of the extracted feature, a tempotron learning rule is used. Tempotron uses the dynamic LIF neuron model which generates a postsynaptic potential for each input spike, which has a fast-rising and slow decaying shape. The number of inputs of the tempotron network is equal to the number of C1 responses. The membrane time constant in the tempotron was set to 0.02, the learning rate was 0.1, and the number of tempotron neurons for each class was 10.

#### Bag of event (BOE)

The BOE (Peng et al., 2016) method is a statistical learning algorithm. BOE is a statistical feature which is used as the representation of each recording. BOE combines two measures of information content. One measurement is the event frequency which is the estimation of the probability that a certain event is actually observed. The other is the speciality measure which reflects the amount of information after observing a specific event. BOE demonstrated a discriminative feature for the combination of the two measurements. Based on the BOE feature, a Support Vector Machine (SVM) classifier was used for the classification of the AER recordings. SVM is a maximum margin classifier by choosing the hyper-plane so that the distance between the hyper-plane and the nearest data point on each side is maximized. In addition, a random forest classifier (Breiman, 2001) was used to classify the BOE representation of the AER recordings. In random forest, an ensemble of trees (5,000 trees in this work) are grown and voted for the most popular class, which results in significant improvements in classification accuracy.

#### Discussion

Table 4 shows that classification results using some statistical features are very close to chance (10% for a 10-class problem). In the statistical classification, feature representation is very important. In general, discriminative features make good predictors of class membership for the classes we are trying to distinguish. For example, having wheels or not distinguishes people from cars, but doesn't distinguish cars from trains. Good classification performance will be achieved when distribution of the statistical feature in one class has little overlap with that in another class. As all the used statistical features are of one dimension, the feature distributions of different classes have much overlap. Moreover, many recordings of different classes may share the same feature values. For example, the mean values of MNoXA for all classes distributes in a relatively larger range than that of MNoYA feature, while the standard deviations of MNoXA for all the classes are larger than that of MNoYA. As such, classification performance using MNoXA is better than using MNoYA. The combination of all the statistics achieved better classification accuracy than the single statistical feature, which demonstrates that it is a more discriminative feature.

**Table 4:** Classification accuracies for CIFAR10-DVS.

**STATISTICS KNN (*K* = 10)**	**Mean accuracy ± std**
Number of events	13.34 ± 0.75%
Number of ON events	12.12 ± 1.03%
Number of OFF events	13.26 ± 0.99%
Ratio of positive events to negative events	11.71 ± 1.02%
Mean of x address	15.01 ± 1.34%
Mean of y address	10.61 ± 0.64%
Standard deviation of x address	14.15 ± 1.03%
Standard deviation of y address	12.70 ± 0.82%
All	16.80 ± 1.10%
SFNN	22.10 ± 0.87%
BOE-SVM	24.21 ± 1.23%
BOE-Random Forest	29.67 ± 1.34%

As shown in Table 4, both SFNN and BOT methods achieve better performance than using statistics of the recordings. However, the classification accuracies using the two methods are also relatively low. The reason is that the two classification methods were designed originally for the easier datasets (i.e., MNIST-DVS). However, the samples of CIFAR10-DVS have more complex objects than digits in MNIST-DVS. As is known, the CIFAR-10 dataset provides a more challenging task than the MNIST task in conventional computer vision. Furthermore, we tried to improve the performance by using random forest classifier on BOE features. When 5,000 trees in the random forest were grown, the classification accuracy was increased to 29.45%. This dataset provides plenty of room for continuous improvements of event-driven algorithms.

Finally, we evaluated the influence of the number of closed loops on the recognition rate and processing time as shown in Table 5. In this evaluation, 1,000 recordings with 100 recordings per class were randomly selected from the CIFAR10-DVS dataset. BOE and SVM based classification method is used in this experiment. Six different numbers of closed loops (i.e., 1, 2, 3, 4, 5, 6) were evaluated with six experiments. In each experiment, 90% of the experimental recordings are used for training and the rest are used as testing set. Each experiment was repeated ten times with different training and testing data partitions. The processing time is the sum of the time cost of feature extraction, training time and testing time. The experiments were carried out on a PC with an Intel(R) Core(TM) i5-3317U-1.7GHz CPU and 8-GB RAM. The final results contain the mean and standard deviation, of the classification accuracies and the processing time. The results show that as the number of closed loops increases, the recognition rate increases while the computational time cost increases as well.

**Table 5:** Recognition and processing time with increasing number of closed loops.

**Number of closed loops**	**Recognition accuracy (mean ± std)**	**Processing time (mean ± std)**
1	16.71 ± 3.87%	201.58 ± 0.51 s
2	18.64 ± 3.83%	424.09 ± 0.89 s
3	19.76 ± 3.15%	642.43 ± 1.24 s
4	20.80 ± 4.13%	865.87 ± 1.61 s
5	21.76 ± 3.22%	1052.9 ± 1.08 s
6	22.70 ± 3.32%	1545.2 ± 2.50 s

## Conclusion

In this paper, we provide a new neuromorphic vision dataset named CIFAR10-DVS for event-based object classification. The conversion of event-stream dataset is implemented using a RCLS movement of the images. The image movement results in the intensity changes in the visual field. The closed-loop image movement in continuous time converts the rich intensity gradients of the image into the intensity changes. The local relative changes of intensity are quantized by the corresponding pixels of the neuromorphic camera to generate event streams. Image movement is easily implemented by programming on computer and is close practical applications. For example, a fixed event-based camera responds only to the moving objects, which reduces the information redundancy. Our recording system can be used to easily convert other large frame-based image datasets with the total time of conversion scaling linearly with the number of images in the dataset. The CIFAR10-DVS dataset will become a data resource for a broad range of event-based or point-based related researches. The possible applications are as follows.

### A benchmark of event-driven object recognition

CIFAR10-DVS provides a more difficult dataset than event-stream recordings of MNIST dataset and a relatively simpler benchmark than N-Caltech101 with fewer categories. It will be a new and challenging benchmark dataset for the event-driven object recognition. The classification accuracies of the three algorithms presented in Section Discussion provide an initial benchmark to improve upon. The classification results on the dataset leave much room for improvement.

### Human vision model research

Each recording of CIFAR10-DVS is captured by a silicon retina which mimics the information coding mechanism of human retina. The all-or-none property of the event is similar to the spike in nervous system. In human vision, visual information is converted by the biological retinas into spikes and processed by the high-level visual cortex. The event-spike datasets may be used as the visual sensing module in the human vision model. For example, the question of how the biological neural network read and process the spike-based information may be researched using the event-spike data. Furthermore, based on neuromorphic cameras, brain-inspired computation and cognition system can be used to process the event-stream data. For example, brain-inspired hardware system can be used to solve the classification task of CIFAR10-DVS.

### A data resource of spatio-temporal point process

In statistics, a point process is a type of random process consisting of a set of isolated points either in time or geographical space. Each event in the event-stream data is like a point in the spatio-temporal point pattern. Point process whose values are point patterns is often used to model many kinds of data, such as spikes of neurons in computational neuroscience (Brown et al., 2004), positions of trees or plants in the forestry and plant ecology. Each event-stream data is a 3-dimensional spatio-temporal event patterns which can be modeled using point process theory. CIFAR10-DVS provides a set of labeled spatio-temporal point patterns. One interesting research direction is to use point process theory to model the spatio-temporal event streams.

## Author contributions

HLi carried out the converting of the dataset and performed the analysis and classification of the dataset. HLiu developed the software system and carried out the converting of the dataset. XJ proposed the research direction and discussed the results. GL took part in the data processing, and discussed the results. LS proposed the research direction and guided the project. HLi and LS drafted the manuscript. All of the authors discussed the results and commented on the manuscript.

### Conflict of interest statement

The authors declare that the research was conducted in the absence of any commercial or financial relationships that could be construed as a potential conflict of interest.

## Footnotes

^1^https://figshare.com/s/d03a91081824536f12a8

^2^http://www2.imse-cnm.csic.es/caviar/MNISTDVS.html

^3^www.garrickorchard.com/datasets
